# BAG3 is involved in neuronal differentiation and migration

**DOI:** 10.1007/s00441-017-2570-7

**Published:** 2017-02-01

**Authors:** Antonietta Santoro, Vanessa Nicolin, Fulvio Florenzano, Alessandra Rosati, Mario Capunzo, Stefania L. Nori

**Affiliations:** 1 Department of Medicine, Surgery and Dentistry, “Scuola Medica Salernitana”, University of Salerno, Salerno, Italy; 2 Clinical Department of Medical, Surgical and Health Science, University of Trieste, Trieste, Italy; 3 Confocal microscopy unit, National Research Council, European Brain Research Institute (EBRI), Rome, Italy

**Keywords:** BAG3, PC12 cells, Neuronal differentiation, Mouse cerebral cortex, Large dense-core vesicles

## Abstract

Bcl2-associated athanogene 3 (BAG3) protein belongs to the family of co-chaperones interacting with several heat shock proteins. It plays a key role in protein quality control and mediates the clearance of misfolded proteins. Little is known about the expression and cellular localization of BAG3 during nervous system development and differentiation. Therefore, we analyze the subcellular distribution and expression of BAG3 in nerve-growth-factor-induced neurite outgrowth in PC12 cells and in developing and adult cortex of mouse brain. In differentiated PC12 cells, BAG3 was localized mainly in the neuritic domain rather than the cell body, whereas in control cells, it appeared to be confined to the cytoplasm near the nuclear membrane. Interestingly, the change of BAG3 localization during neuronal differentiation was associated only with a slight increase in total BAG3 expression. These data were coroborated by transmission electron microscopy showing that BAG3 was confined mainly within large dense-core vesicles of the axon in differentiated PC12 cells. In mouse developing cortex, BAG3 appeared to be intensely expressed in cellular processes of migrating cells, whereas in adult brain, a diffuse expression of low to medium intensity was detected in neuronal cell bodies. These findings suggest that BAG3 expression is required for neuronal differentiation and migration and that its role is linked to a change in its distribution pattern rather than to an increase in its protein expression levels.

## Introduction

Bcl2-associated athanogene 3 (BAG3) belongs to the family of co-chaperones interacting with the ATPase domain of the heat shock protein HSP70 through the structural domain known as the BAG domain. BAG3 has a modular structure also containing a WW domain and a proline-rich region (PxxP) that mediates the binding to other proteins including the SH3 domain containing phosholipase C-γ (PLCγ) and the minus-end directed microtubule motor dynein (Arndt et al. 2010; Gamerdinger et al. 2011; Rosati et al. 2011). Two other conserved IPV (Ile-Pro-Val) motifs have been identified; they are responsible for the interaction of BAG3 with the small heat shock protein HSPB8. The HSPB8-BAG3 complex is able to induce macroautophagy and assist the autophagy-mediated clearance of misfolded proteins including poliQ-expanded huntingtin and mutated SOD1 proteins (Carra et al. 2008a; Gamerdinger et al. 2011). Interestingly, BAG3 has been reported to mediate the retrograde transport of both misfolded proteins to the microtubule organizer center (MTOC) and autophagic vacuoles to the perinuclear region, thus facilitating the clearance of aggregated-prone proteins (Ravikumar et al. 2005; Carra et al. 2008b). In addition, in rat primary neurons, BAG3 overexpression has been demonstrated to result in a significant increase in the degradation of tau protein, a key neuronal protein regulating cytoskeletal dynamics and synaptic function and implicated in the pathogenesis of Alzheimer’s disease (Lei et al. 2015). In the central nervous system (CNS), BAG3 protein has been detected in reactive astrocytes from HIV-1-associated encephalopathy biopsies and in human glial cells and its expression appears to be modulated by several stress-inducers including hypoxia-ischemia and viral infection (Rosati et al. 2007; Bruno et al. 2014a). Seidel et al. (2012) demonstrated that BAG3 expression levels are upregulated in astrocytes from human brain tissues of patients with Alzheimer’s, Parkinson’s, or spinocerebellar ataxia type 3 (SCA3) disease, whereas no significant variation of HSPB8-BAG3 expression has been found in neurons of the same patients (Seidel et al. 2012). Of interest, BAG3 has been observed to increase in neurons of rodent brain during aging, together with an enhanced expression of autophagosomal marker LC3-II (Gamerdinger et al. 2009). These findings suggest that BAG3 is involved in neurodegenerative disease progression and its overexpression could be required during aging to compensate partially for the reduced capacity of the proteasomal-mediated degradation of misfolded proteins (Keller et al. 2004; Li and Li 2011).

However, to date, only a few studies have addressed BAG3 distribution and function in the CNS. These studies have shown that the protein presents a selective tissue distribution pattern and that its expression, at the tissue and cellular levels, is modified during development. Among them, some papers reported BAG3 expression in rat CNS with a persistent expression in the rostral migratory stream and the subventricular zone of the lateral ventricle and both a rapid and transient increase of BAG3-positive neurons in the cortex and hippocampus during the first postnatal week (Choi et al. 2006). More recent results indicate that BAG3 is expressed in adult rat hippocampal neural progenitors in response to fibroblast growth factor 2 (FGF2) treatment (Gentilella and Khalili 2010) and an early transient expression of BAG3 has been observed in radial glia in the developing brainstem and spinal cord of rats (Choi et al. 2009). However, no data have been reported on the subcellular distribution of BAG3 in neurons, although we have detected a molecular weight variant of BAG3 (40 kDA) in synaptosomes of rat brain and provided evidence that BAG3 mRNA is present in synaptosomal polysomes (Bruno et al. 2014b). Taken together, these considerations suggest that BAG3 is involved in several specific neural functions and participates in neuropathological processes. Therefore, in order to provide further insights into the role of BAG3 in neuronal differentiation and migration, we studied BAG3 localization and expression in nerve growth factor (NGF)-induced neurite outgrowth in the PC12 cell model and examined the expression of BAG3 in the developing and adult cerebral cortex of mice.

## Materials and methods

### Cell culture and NGF treatment

Rat pheochromocytoma PC12 cells were maintained in RPMI 1640 Medium (Invitrogen, Italy) supplemented with 10% horse serum (Invitrogen) and 5% fetal calf serum (Invitrogen) and grown as monolayer cultures on Falcon dishes in a humidified atmosphere at 37 °C and under 5% CO_2_. To induce the differentiation of PC12 cells into neurons, cells were plated at a concentration of 2 × 10^5^/dish (100 mm plates, BD Falcon) and cultured for 12 h. The cells were then divided into two groups: the first did not receive treatment (untreated, controls), whereas the second was treated with 100 ng/ml NGF (Sigma, St Louis, Mo., USA). Cells received a medium change every 2 days and were treated every day with the same NGF concentration as previously described (Levi-Montalcini 1987; Arisi et al. 2014). To check cultures for their differentiation degree, light microscope images of the living differentiating cells were taken and neurite length was evaluated. Only cultures presenting cells with a neuritic length of at least 20 μm were destined for imaging procedures. On the basis of the degree of differentiation, we chose to perform immunofluorescence after 5 days of NGF treatment. After two washes in phosphate buffer (PB), cells were incubated in 4% paraformaldehyde/PB (Sigma) for 20 min followed by three washes in PB.

### Animals and histological procedures

The following animals were used: three adult male mice (C57BL/6 N; Harlan-Nossan, Correzzana, Italy) and three mice at embryonic day 18 (E18; the day of vaginal plug discovery in pregnant mothers was considered E0). Animal care and experiments were conducted in conformity with Italian and international laws and policies (EEC Council Directive 86/609, OJ L 358,1,12 December 1987; NIH Guide for the Care and Use of Laboratory Animals, NIH Publication no. 85–23, 1985). The adult animals were transcardially perfused under deep anesthesia (sodium pentobarbitol, 60 mg/kg, i.p.) with 150 ml 0.9% saline at room temperature (RT) followed by 200 ml 4% paraformaldehyde in 0.1 M PB at pH 7.4 (PafPB). Brains were dissected, post-fixed for 2 h at room tempertaure and after three washes in PB for 10 min, cryoprotected in 30% PB/sucrose at 4 °C. Embryonic pups were removed from the mothers, anesthetized by immersion in dry ice and killed by decapitation followed by dissection of their brains. Brains derived from embryonic pups were immersed in PafPB for 24 h and from postnatal animals for 48 h. After three washes in PB of 10 min each, brains were cryoprotected in 30% sucrose/PB at 4 °C. Once the brains had soaked, they were frozen in dry ice and cut into 40-μm-thick transverse sections on a sliding microtome. For adult animals, one in every 10 sections was collected in PB, whereas for embryonic and postnatal animals, one in every three sections was collected for free-floating immunofluorescence procedures. Qualitative inspections were made of all the major brain regions, whereas quantitative observations were confined to the cerebral cortex. Boundaries and subdivisions were identified with reference to the atlas of Paxinos and Watson (2005).

### Immunofluorescence and confocal microscopy

The following antibodies were used: mouse monoclonal anti-BAG3 (1:400; AC-1, kindly provided by BIOUNIVERSA, Salern, Italy); rabbit anti-β-tubulin III (1:200; Sigma); rabbit monoclonal anti-neuronal nuclei (1:500; NeuN; Millipore) for both tissue sections and cell cultures. Tissue sections were incubated for 2 days at 4 °C in 0.3% Triton X-100/PB (PBT) with a mix of the following primary antibodies: AC-1/β-tubulin or AC-1/NeuN. After being washed in PBT, the sections were incubated for 2 h at RT with Alexa-Fluor-488 donkey anti-rabbit IgG (1:200) and Alexa-Fluor-555 donkey anti-mouse IgG (1:200).

Cell cultures were preincubated for 10 min in 0.1% PBT and incubated overnight at 4 °C with a mix of the following primary antibodies: AC-1/β-tubulin. After being washed with PB, the cells were incubated for 1 h at RT with Alexa-Fluor-488 donkey anti-rabbit IgG (1:500) and Alexa-Fluor-555 donkey anti-mouse IgG (1:600). After two rinses in phosphate-buffered saline (PBS), cells were treated for 10 min with a 4′,6-diamidino-2-phenylindole (DAPI; 1:500) solution (Molecular Probes, Invitrogen) for visualization of nuclei.

Controls of both sections and cell cultures were processed as above, except that the primary antibody was omitted or substituted with nonspecific rabbit IgG. Both sections and cell cultures were mounted on slides, coverslipped with mounting gel and examined under a confocal laser scanning microscope (Leica SP5, Leica Microsystems, Germany) under sequential mode to avoid crosstalk among channels. Confocal image acquisition was conducted so that all samples were imaged by using consistent settings for pinhole aperture, laser power and detector gain. Image processing and the preparation of the final figures were carried out by using Adobe Photoshop 7 and Adobe Illustrator 10.

### Image analysis

Image analysis of anti-BAG3 (AC-1) confocal immunofluorescence was performed by Imaris 7.4 (Bitplane) software as previously described (Amadoro et al. 2014) on five different images derived from three different cell culture replicates for each experimental group. By using the transmitted light images showing the cellular profiles and those merged with the immunofluorescence signal, a mask was manually drawn on cells. Therefore, all the parameters were selectively evaluated only on cellular profiles. Image analysis was performed under visual control to determine thresholds; background noise was subtracted and neuronal and vesicular structures were taken into account. During image processing, the images were compared with the raw data to make sure that no structures were introduced or removed from the original data series.

Morphological parameters under analysis included immunofluorescence intensity, number, area and colocalization of vesicle-like structures, which were evaluated by using Surface, Spots, and Coloc Imaris modules. To discriminate between inner and outer cytoplasmic domains, another mask was imposed on the cytoplasm and approximately divided the cell into two halves: one juxtaposed to the cell membrane and the other juxtaposed to the nucleus. To determine fluorescent signal colocalization between the different channels, a median filter was applied to reduce the background noise and the degree of overlap was measured by Pearson’s coefficient, which describes the relationship between the pixel intensities of two channels by linear regression as previously described (Nori et al. 2013).

### Western blot analysis

Immunoprecipitation and Western blot analysis were performed as previously described (Santoro et al. 2009; Bruno et al. 2014a, 2014b). PC12 cells untreated or treated with NGF were collected by centrifugation, washed twice with PBS and resuspended in RIPA buffer (NaCl 150 mM, 1% Triton X-100 pH 8.0, 0.5% sodium deoxycholate, 0.1% SDS, 50 Mm TRIS, pH 8.0) and 1× protease inhibitor cocktail (Sigma) for 30 min on ice. Lysates were centrifuged at 4 °C and 15,000 rpm for 30 min. Supernatants were collected and the protein concentration was determined by the Bradford protein assay. Equal amounts of protein extracts (40 μg) were boiled in Laemmli’s buffer, fractionated by 10% SDS-polyacrylamide gel electrophoresis and then transferred to nitrocellulose membranes (Amersham, GE Healthcare). Membranes were blocked in TBST (20 mM TRIS–HCl pH 7.4, 500 mM NaCl and 0.01% Tween-20) containing 10% non-fat dry milk, washed in TBST and then incubated overnight at 4 °C with mouse monoclonal anti-BAG3 (1:1000; AC-1). Anti-GAPDH (D-glyceraldehyde-3-phosphate dehydrogenase) was used as loading control (Cell Signalling). Blots were probed with mouse or rabbit secondary antibodies for 1 h and then developed by using an enhanced chemiluminescence (ECL) system (Amersham, GE Healthcare). Western analysis was performed at least three times and representative results are shown.

### Electron microscopy

A cell monolayer was sequentially dehydrated in ascending alcohols and infiltrated for 4 h at RT with three changes of LR white acrylic resin hard (Sigma). Coverslips were covered with a cylindrical capsule filled with fresh LR white resin and polymerized at 60 °C for 24 h. Thin sections were collected on 400-mesh nickel grids, incubated for 30 min at RT in TBS blocking buffer (20 mM TRIS–HCl, pH 7.6, 225 mM NaCl and 1% bovine serum albumin) containing 5% normal goat serum and then incubated overnight at 4 °C with a polyclonal antibody recognizing primary monoclonal antibody (AC-1, 8 ng/ml). After a 10-min washing step in TBS blocking buffer, samples were incubated for 1 h at RT with EM goat anti-mouse IgG 10 nm gold. Control grids were also included in which the primary antibody was omitted. Sections were stained with uranyl acetate and examined by means of a JEOL 100S electron microscope (JEOL, Peabody, Mass., USA) operating at 80 kV.

### Statistical analysis

Data were analyzed by GraphPad 5 software (GraphPad Software, USA). Image analysis data were expressed as means ± standard deviation (SD). Statistical differences between the treatments and the controls were evaluated by one-way analysis of variance (ANOVA). In the case of a significant result in the ANOVA, a two-tailed Student’s *t* test was performed. Differences were considered statistically significant when *P* < 0.05.

## Results

### BAG3 localization in differentiated PC12 cells

**Fig. 1:**
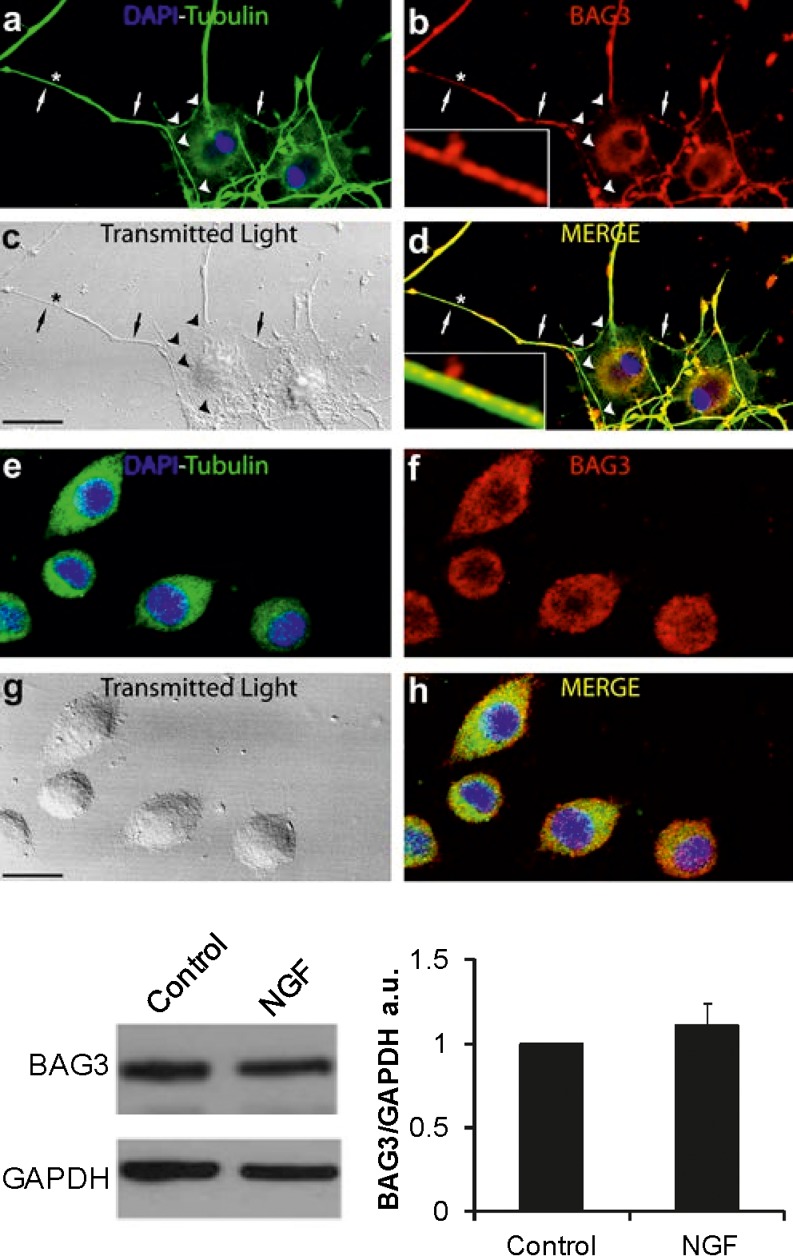
*Top* Representative photomicrographs (**a–h**) of Bcl2-associated athanogene 3 (*BAG3*) expression in PC12 cells after nerve growth factor (NGF) priming (**a–d**) and in the basal condition (**e–h**). Confocal and transmitted light (*gray channel*) images of double immunofluorescence for BAG3 (*red channel*), β-tubulin III (*green channel*) and 4′,6-diamidino-2-phenylindole (*DAPI*; *blue channel*). Note regions containing BAG3-positive, loosely packed, granular structures (*arrows*, see also *inset*) or barely detectable BAG3 immunopositivity (*arrowheads*). *Insets* Neuritic region marked with an *asterisk* in **a–d**. *Bars* 15 μm. *Bottom left* Representative Western blot of BAG3 in PC12 cells untreated (*Control*) or treated with NGF for 5 days (*NGF*). D-glyceraldehyde-3-phosphate dehydrogenase (*GAPDH*) was used as a loading control. *Bottom right* Densitometric analysis showing means in arbitrary units (*a.u.*) and SD from three independent experiments

In order to investigate the localization and expression of BAG3 protein during neuritic formation, we chose a cell model system partially resembling neuronal differentiation phenomena. We used pheocromocytoma cells (PC12), a NGF-responsive cell line derived from rat adrenal medulla. This cell line is known to stop mitotic division and terminally differentiate when treated with NGF (Levi-Montalcini 1987). Immunofluorescence studies were performed both in NGF-treated and NGF-untreated cultures. As shown in Fig. 1, after NGF-induced neurite outgrowth, BAG3 localized in the neuritic domain (Fig. 1a–d) and most of the immunoreactivity seemed to be confined in granules resembling vesicular structures. In undifferentiated PC12 cells, BAG3 protein was expressed in the cell body (Fig. 1e–h) and the immunoreactivity appeared to be densely packed and homogeneously distributed in the cytoplasm. From a qualitative point of view, in NGF-stimulated cells, BAG3 expression in the cell body appeared to be lower compared with that in untreated cells and was located in the inner cytoplasmatic region, being virtually absent in the outer cytoplasmatic region. In addition, in the neuritic domain of NGF-stimulated cells, BAG3 expression was higher than in the corresponding cell bodies. The BAG3 immunoreactivity densely filled the neuritic domains and colocalized with β-tubulin III, although a closer inspection of higher magnification images (see insets in Fig. 1b, d) showed that BAG3-immunopositive and β-tubulin-III-negative dots were also evident suggesting a vesicular nature of BAG3-immunopositive granules. This peculiar pattern distribution suggests that, following BAG3 production, the protein is immediately destined for the neurites where it shows the highest expression.

To ascertain whether BAG3 expression could change during NGF treatment, Western blot analysis was performed in PC12 cells after the same treatment time and with the same NGF concentration as used in the immunofluorescence analysis. The results (Fig. 1, bottom) indicated that no significant variation of the BAG3 expression level occurred during PC12 cell differentiation, thus indicating that the subcellular localization of the protein changed during neuronal differentiation but with no substantial increase in the protein expression level.

**Fig. 2:**
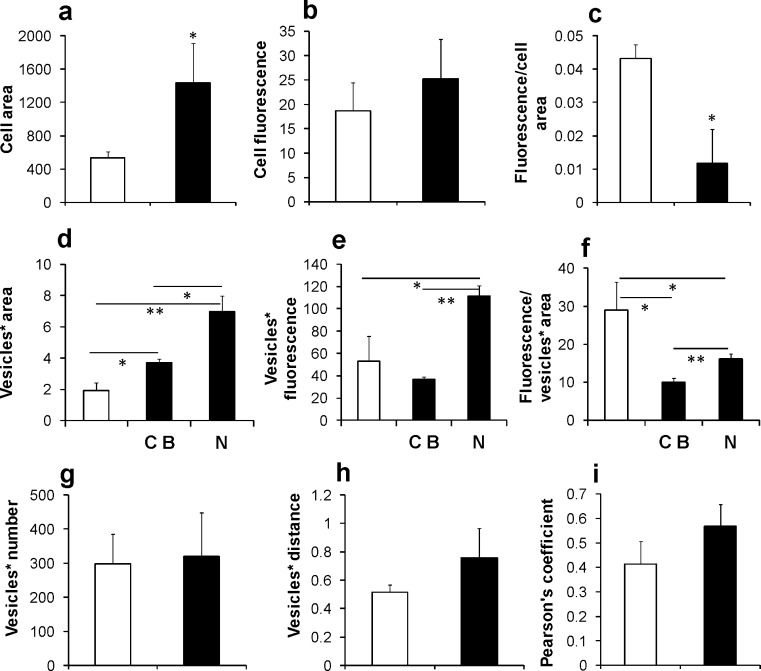
Image analysis of BAG3 confocal immunofluorescence in PC12 cells untreated (*white bars*) or treated (*black bars*) with NGF for 5 days. Data are reported as means and SD of three independent experiments. *P < 0.05; **P < 0.01 with the Student’s *t* test. **a–c** Evaluation of BAG3 expression in total cell area of untreated and NGF-primed cells. **d–f** Evaluation of proportion of the vesicle-like structures (Vesicles*) expressing BAG3 in cell bodies (*CB*) and neurites (*N*). **g**, **h** Estimation of the immunopositive vesicle-like structures (Vesicles*), namely their number and their mean distance (μm) from the cell membrane in untreated and NGF-treated cell bodies. **i** Pearson’s coefficient was used as a colocalization index of β-tubulin III and BAG3; values from 0 to 0.5 indicate absence of colocalization, whereas values from 0.6 to 1.0 indicate colocalization

On the basis of the previous results, we estimated the different contributions of the segregated and total fraction of BAG3 in the various cellular domains by performing morphometric analysis of the total fluorescence and the fluorescence of dots resembling vesicle-like structures, either in cell bodies or in neuritic domains (Fig. 2). First, we measured the total immunopositive cell area and fluorescence intensity in both NGF-treated and untreated cells. As shown in Fig. 2a, b, stimulation with NGF significantly increased the mean cell area (166%), whereas only a slight and non-significant increase was observed in cell fluorescence intensity in NGF-treated cells compared with the controls (45%). Indeed, following neuronal differentiation, the cell area, the vesicle-like structure area and the vesicle number were different from the basal condition, thus affecting the quantification of BAG3 expression by immunofluorescence. Therefore, we normalized the fluorescence intensity against the measured cell area (Fig. 2c) and the results showed that the mean amount of immunofluorescence for cell area was higher in the control NGF-untreated cells (72%) compared with treated cells (Fig. 2c vs a, b). This finding suggests that the observed increase of cell fluorescence in NGF-treated cells is mainly attributable to the neuritic domain.

Moreover, the separate evaluation of the vesicular-like structures area and their fluorescence intensity, in both cellular and neuritic domains of NGF-treated cells, showed a significant increase of the mean area of these vesicular structures in the cell bodies of NGF-stimulated cells versus controls (93%) and in the neuritic domain versus the cell body domain of NGF-treated (88%) and control (264%) cells (Fig. 2d). Data concerning the fluorescence intensity of vesicle-like structures in NGF-treated cells (Fig. 2e) showed a reduction of BAG3 fluorescence intensity in the cell bodies, together with an increase in vesicle-like structure fluorescence of BAG3 in the neuritic portion, suggesting that the expression of BAG3 was higher in the neutites. Normalization of the fluorescence intensity with the area of vesicle-like structures (Fig. 2f) revealed that the detected protein intensity enhancement was related to an increase in the area of neuritic granules. These data obtained by quantitative image analysis were also in agreement with Western analysis.

In cell bodies of the NGF-treated cells, BAG3 expression was distributed particularly within the inner cytoplasmic domain (Fig. 1) leaving the outer cytoplasmic domain almost completely devoid of BAG3 immunofluorescence. We quantified this peculiar spatial distribution by evaluating the mean number of immunopositive vesicle-like structures and their distance from the cell membrane in NGF-treated and untreated cell bodies. Measures reported in Fig. 2 (Fig. 2g, h) indicate a general increase of the mean distance (46%) of BAG3-immunopositive vesicular structures in NGF-stimulated cells associated only with a modest increase of their mean total number (7%). This result suggests that, also in the cell body, BAG3 expression does not drastically change following NGF-treatment but is redistributed. Although BAG3 and β-tubulin were visually intimately associated (Fig. 1), the colocalization index (Pearson’s coefficient, Fig. 2i) was not high in the two groups. However, NGF-primed PC12 cells showed a higher Pearson’s correlation coefficient with respect to the control group indicating that BAG3 is also associated with the cytoskeleton.

### BAG3 localization in large dense-core vesicles

**Fig. 3:**
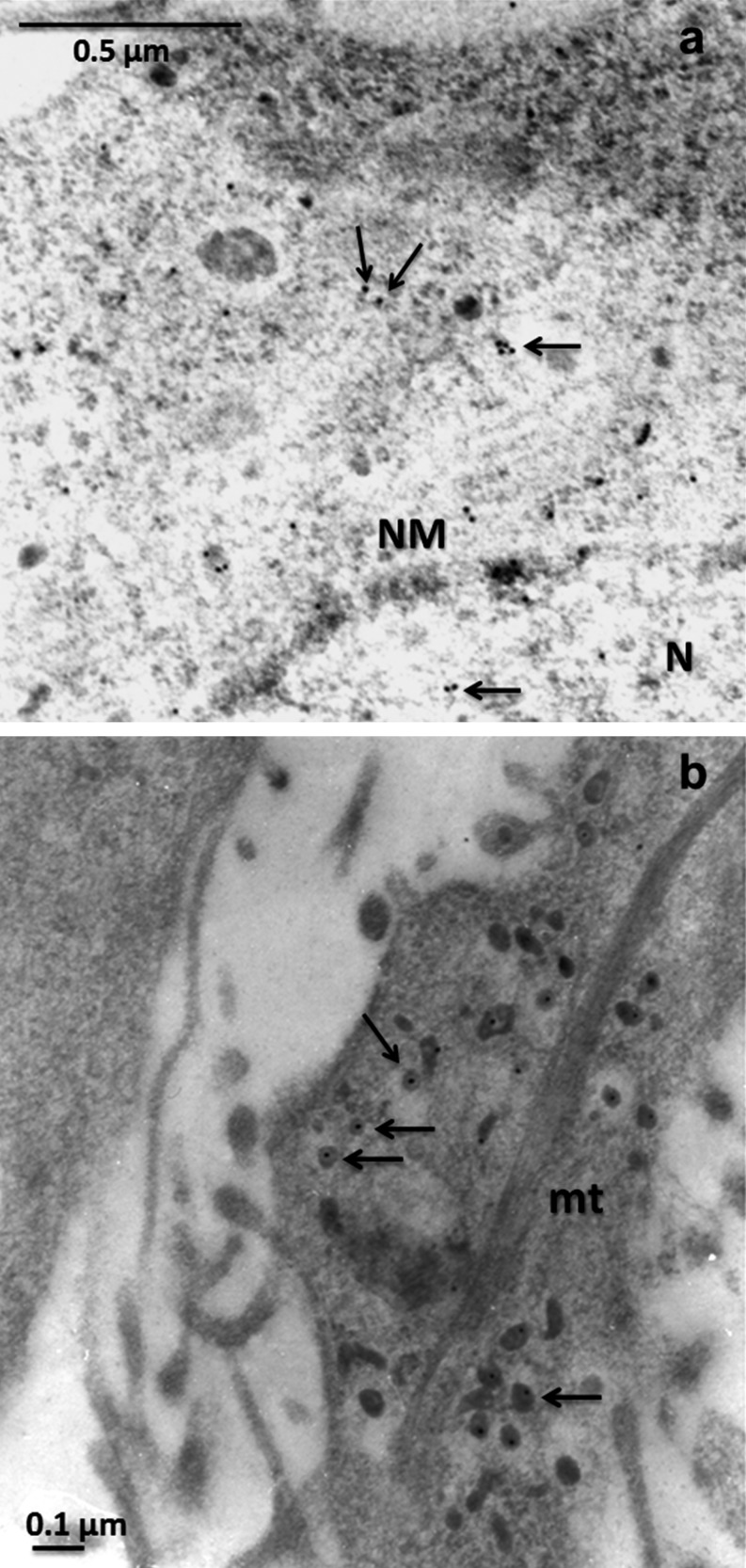
Transmission electron micrographs of NGF-induced PC12 cell differentiation showing localization of BAG3 (*arrows*). **a** Representative micrograph of cell body (*N* nucleus, *NM* nuclear membrane). *Bar* 0.5 μm. **b** Representative micrograph of the cell axon (*mt* mitochondrion). *Bar* 0.1 μm. PC12 cells showed rare BAG3 immunogold-labelled (10 nm) positivity in the nucleus and nucleolar region (**a**), whereas BAG3 appeared more marked in the cytoplasm and in large dense-core vesicles of the axon (**b**)

To investigate further the intriguing expression of BAG3 protein in NGF-induced PC12 cell differentiation, we analyzed the subcellular localization of BAG3 by transmission electron microscopy (TEM). After treatment with NGF, as carried out for confocal microscopy analysis, cells were immunoreactive to BAG3 antibody and BAG3 appeared to be distributed at different morphological levels in the cell body (Fig. 3), whereas in negative controls, no label was seen (data not shown). As revealed in Fig. 3a, BAG3 was diffuse inside the nucleus and in transition to the cytoplasm and was particularly detectable inside the inner cytoplasmic areas with a granular texture. Furthermore, BAG3 localized clearly into electron-dense vesicles clustered along the axon (Fig. 3b); these BAG3-positive vesicles showed the typical aspects of large dense-core vesicles (LDCVs).

### BAG3 localization in developing and adult mouse brain

**Fig. 4:**
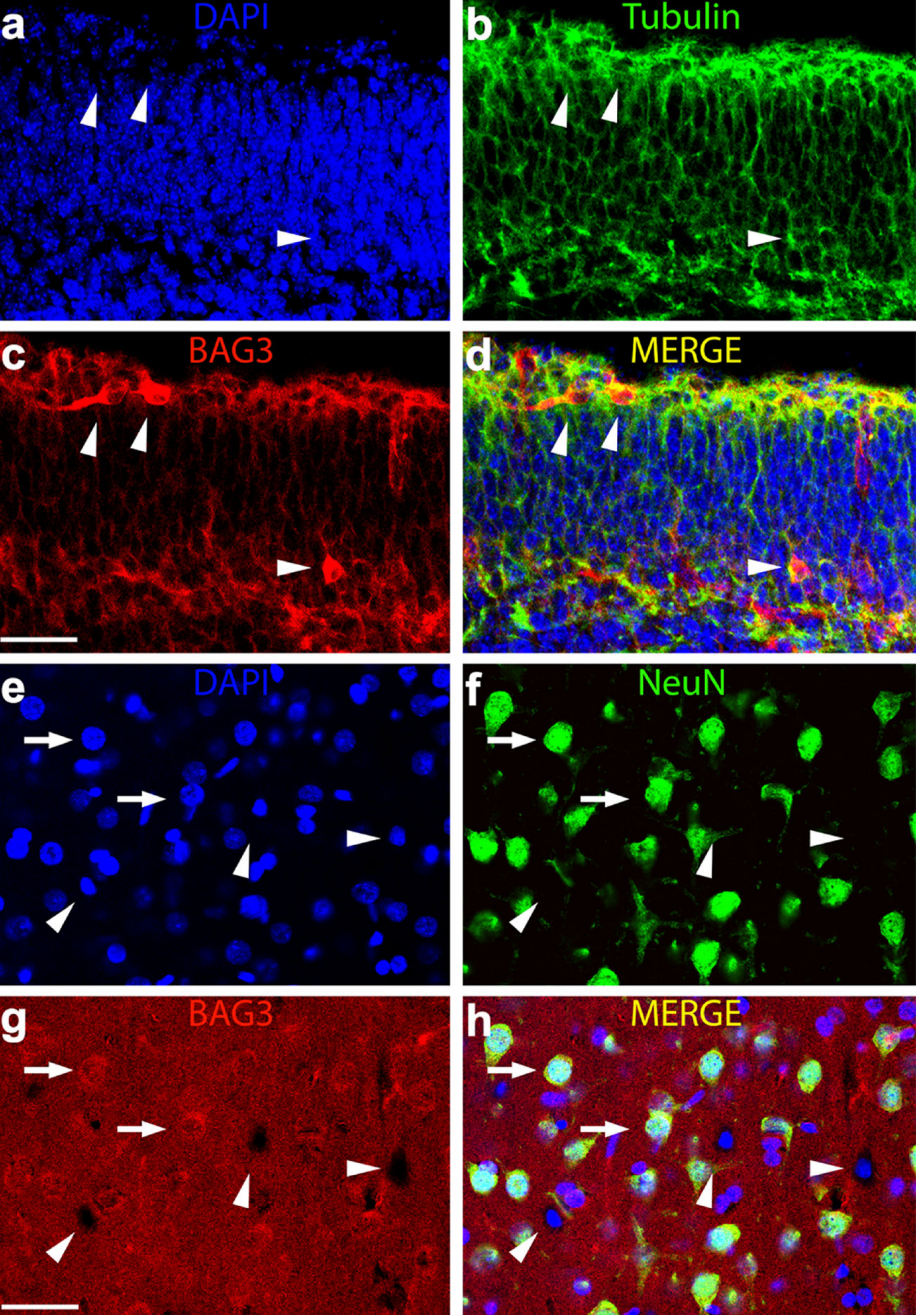
BAG3 expression in the developing (E18, **a–d**) and adult cortex (2 months, **e–h**) of mouse brain. Confocal images of double immunofluorence for BAG3 (*red channel*), β-Tubulin III (**a–d**, *green channel*), NeuN (**e–h**, *green channel*) and DAPI (*blue channel*). BAG3 in the developing cortex is intensely expressed in cellular processes and some migrating cells (*arrowheads*). In the adult cortex, low to medium immunoreactivity is mainly found in the majority of neuronal cell bodies (*arrows*) and in some glial cells (*arrowheads*) identified as non-NeuN-positive. *Bars* 30 μm (**a–d**), 40 μm (**e–h**)

Since BAG3 expression was shown to be associated with NGF-induced neuronal differentiation in PC12 cells, we decided to investigate its expression in the developing (E18) and adult brain. In the embryonal brain, BAG3 immunoreactivity in the cortex was mainly distributed in the ventricular and marginal zones where it appeared localized to filamentous structures organized in both the horizontal and the vertical planes (Fig. 4a–d). Of note, several cells displayed intense BAG3 immunofluorescence in the cell bodies and proximal processes, suggesting that at least a part of the fluorescence signal was ascribable to the cellular processes of cells that do not reside in the confocal image acquisition plane. Some of these intensely stained cells exhibited the typical bipolar morphology of migrating cells (Nadarajah et al. 2003; Garcez et al. 2015), with the cell body position and orientation either on the tangential plane or on the radial plane and with a straight and thin leading process.

In the adult cortex, however, we found a medium to low diffuse expression of BAG3, both in NeuN-positive and NeuN-negative cells (Fig. 4e–h), thus indicating the expression of BAG3 in neurons but probably also in glial cells. This distribution pattern appeared to be widespread throughout the brain with only slight regional variations.

## Discussion

In the present study, we assessed the role of BAG3 in neuronal development and showed that BAG3 is redistributed during neuronal differentiation in vitro and is overexpressed during neuronal migration in vivo. BAG3 protein is a member of the BAG family, which plays a central role in cell protein quality control and ubiquitin proteasome systems, two major pathways that, together with the lysosomal degradation pathway, are referred to as undertaking macroautophagy and that regulate protein homeostasis and cytoskeletal remodeling (Gamerdinger et al. 2011). To date, the increasing literature has reported that BAG3 overexpression in glioma cells results in the stimulation of autophagy (Merabova et al. 2015), whereas in osteosarcoma and melanoma cells, it promotes cell survival by sustaining the activation of NF-κB and preventing IKKγ degradation (Ammirante et al. 2010; Rosati et al. 2011). These observations suggest a complex regulative mechanism of this protein that, because of its capacity to interact with very different client proteins, could be involved in a variety of biological processes from the balance of autophagy/apoptosis to cell proliferation and differentiation. However, few data are available concerning the physiological role of BAG3 in the nervous system. In order to analyze BAG3 expression and localization during neuronal differentiation, we used the rat pheochromocytoma cells PC12, which are a well-known established in vitro model for studying neuronal differentiation, proliferation and survival (Greene and Tischler 1976; Levi-Montalcini 1987). After stimulation with NGF, these cells differentiate into neuron-like cells, which are morphologically identifiable by cell hypertrophy and by the appearance of long neuritic processes of various calibers organized in a network.

Under our experimental conditions, dividing and non-NGF-stimulated cells showed marked expression of BAG3 in densely packed granules of the cytosolic fraction, whereas in NGF-treated cells, BAG3 appeared to be localized mainly in vesicular-like structures that filled the neuritic domain. Indeed, a medium to low immunofluorescence intensity of BAG3 was also evident in the inner cytoplasm of NGF–primed PC12 cells, suggesting that the protein might be stored in cytoplasmic vesicles that thereafter migrate into neurites. Morphometric and Western blot analyses of BAG3 expression corroborated this hypothesis, since the total amount of BAG3 immunofluorescence and the protein expression level were not increased in NGF-treated cells compared with controls. On the contrary, BAG3 immunoreactivity filled neurites and increased in dots of the neuritic domain, being also partially associated with β-tubulin, thus suggesting that BAG3 is located in vesicles travelling along the cytoskeleton and is required to control the correct folding of neurite-specific proteins and/or to direct protein-protein interactions during neuritic formation. From this standpoint, our findings are in agreement with the results of Lei et al. (2015) who provided evidence of a tight correlation between the level of the tau protein, which is involved in cytoskeletal dynamics and synaptic function and BAG3 expression in rat primary neurons. Moreover, a physical interaction between cytoskeleton–regulating proteins and BAG proteins has also been reported (Ravikumar et al. 2005; Carrettiero et al. 2009). Of note, in some cases, BAG3-positive beads could be identified in our confocal microscopy images. Neuritic beads are thought to represent degenerative structures in which the cellular cytoskeleton is collapsing (Takeuchi et al. 2005); however the precise physiological significance of neuritic beading is still unclear. One possibility is that BAG3 could have more than one role, i.e., it may control protein folding and interaction during neuritic formation and might stimulate the authophagy process to favor the degradation of defective proteins in degenerating or remodeling cytoskeletal structures. This is in agreement with previously reported results showing that BAG3 regulates macroautophagy pathways and is required in stress conditions and during aging (Carra et al. 2008a, 2008b; Gamerdinger et al. 2009). Interestingly, our TEM analysis confirmed our findings obtained by confocal microscopy and clearly show the localization of BAG3 in the perinuclear region and in LDCVs of the axon. LDCVs are known to store cathecolamine in PC12 cells (Dong et al. 2014) and therefore, we can hypothesize that BAG3 also plays a role in catecholamine storage. We also found a weak BAG3 positivity inside the nucleus of differentiated PC12 cells but the functional and molecular significance of this finding remains to be explored.

In our study, we also analyzed BAG3 expression and distribution in the cortex of mouse brain. We showed that, in E18 embryos, BAG3 immunoreactivity was localized particularly at the level of the ventricular and marginal zone in neurons showing the typical features of migrating cells (Nadarajah et al. 2003; Garcez et al. 2015), whereas in the adult brain, a lower BAG3 intensity appeared widespread in neuronal cell bodies and in the neuropil. Of note, by using NeuN as a marker of neurons, we showed that BAG3 was expressed not only in neurons but also in glial cells identified as NeuN-negative cells. This finding suggests that the protein is expressed not only in astrocytes as previously reported (Seidel et al. 2012) but also in oligodendrocytes and microglia. However, this issue needs further investigations. Our in vivo data are also in agreement with previous results obtained by Choi et al. (2006) who provided evidence of BAG3 immunoreactivity in the cerebral cortex of rat embryos at the E18 stage and a reduction of BAG3 expression starting from the 7th postnatal day (P7). The expression of BAG3 was also studied by Park et al. (2003) who demonstrated selectively BAG3-labeled cells in the subventricular zone of the lateral ventricle of the adult rat brain (Park et al. 2003). However, to analyze BAG3 distribution, these authors used a different primary antibody (rabbit polyclonal anti-Bis antibody) detecting BAG3 protein at its known molecular weight of 75 kDa. In our study, we used a highly specific mouse monoclonal antibody (AC-1) that was raised against a BAG3 N-terminal peptide and that also recognizes different BAG3 protein species with a lower molecular weight. From this point of view, Bruno et al. (2008) previously demonstrated that, by using the traditional BAG3 polyclonal antibody, a clear signal in total (cortex + hippocampus) rat homogenate and in several subcellular fractions can be observed, whereas the BAG3 signal is much less evident in the synaptosomal fraction. However, when the same authors used the AC-1 monoclonal antibody (the antibody that we used), they were able to isolate BAG3 protein in synaptosomes, thus demonstrating that, in the synaptosomes of rat brain, the shorter 40-kDa form of BAG3 possessing the N-terminal region but lacking the C-terminal end is abundant. From this viewpoint, the general more widespread CNS distribution that we found compared with other authors might be attributable to the expression of the different BAG3 forms recognized by our antibody. This was also corroborated by the observation that, in E18 and adult brain of rats, we obtained highly comparable results by using the same antibody (data not shown). Moreover, we have to underline that Bruno et al. (2008) also provided evidence that the shorter 40-kDa form of BAG3 seems particularly expressed in cortex homogenates and we have demonstrated elsewhere that it is possibly traduced by a dedicated mRNA also present in synaptosomal polysomes of rat brain (Bruno et al. 2014a). On the basis of our microscopic analysis, we cannot establish which form is predominant in neurons or whether a differential expression of the BAG3 forms occurs in cell bodies or neurites. However, in the present work, we demonstrated that, in the adult cerebral cortex, BAG3 expression appears low but more widespread than was previously thought and that this expression is compatible with a role for BAG3 in many different neural functions. In addition, the finding that, in the embryonic cerebral cortex, BAG3 is more intensely expressed suggests a role in neuronal migration and differentiation.

In conclusion, our present findings, together with previously reported results, open new intriguing questions concerning the possibility that the presence and differential expression of BAG3 forms have other complementary or alternative functions. Moreover, both BAG3 forms could be required for the correct development of the nervous system and for the maintenance of protein homeostasis.

## Abbreviations

BAG3: Bcl2-associated athanogene 3
CNS: Central nervous system
FGF: Fibroblast growth factor
HSP: Heat shock protein
LDCV: Large dense-core vesicle
MTOC: Microtubule organizer center
NGF: Nerve growth factor
PLCγ: Phosholipase C-γ
SCA3: Spinocerebellar ataxia type 3
SOD: Superoxide dismutase
TEM: Transmission electron microscopy
